# The Treatment of Pleurisy

**Published:** 1875-03

**Authors:** 


*Treatment of Pleurisy*, with an appendix of cases showing the value of combinations of Croton Oil, Ether, and Iodine as counter-irritants in other diseases. By John W. Corson, M. D. Wm. Wood & Co., New York, Publishers.

